# Endometriosis: A Comprehensive Exploration of Inflammatory Mechanisms and Fertility Implications

**DOI:** 10.7759/cureus.66128

**Published:** 2024-08-04

**Authors:** Sachin Rathod, Amardeep Shanoo, Neema Acharya

**Affiliations:** 1 Obstetrics and Gynaecology, Jawaharlal Nehru Medical College, Datta Meghe Institute of Higher Education and Research, Wardha, IND

**Keywords:** reproductive health, disease pathogenesis, immune dysregulation, fertility implications, inflammatory mechanisms, endometriosis

## Abstract

Endometriosis is a prevalent gynecological disorder characterized by the ectopic growth of endometrial-like tissue outside the uterus. This condition poses significant challenges due to its chronic nature, debilitating symptoms such as pelvic pain and infertility, and substantial impact on quality of life. Central to the pathogenesis of endometriosis are inflammatory mechanisms that perpetuate tissue proliferation, adhesion formation, and immune dysregulation within the pelvic cavity. Inflammation plays a pivotal role in the development and progression of endometriosis, influencing the severity of symptoms and complications associated with the disease. Dysregulated immune responses contribute to the persistence of ectopic endometrial implants, exacerbating pelvic pain and other symptoms experienced by affected individuals. Moreover, the inflammatory milieu created by endometriotic lesions disrupts normal ovarian function, impairs follicular development, and compromises reproductive outcomes, thereby posing challenges to fertility. This review comprehensively explores the inflammatory mechanisms underlying endometriosis and their implications for fertility. Synthesizing current research and clinical insights elucidates the intricate interplay between inflammation, disease progression, and reproductive health outcomes. Understanding these complex interactions is essential for developing targeted diagnostic strategies and optimizing therapeutic approaches tailored to alleviate symptoms and improve fertility outcomes in individuals with endometriosis. Ultimately, this review aims to enhance the understanding of endometriosis pathophysiology, inform clinical practice, and stimulate further research to advance personalized care and management strategies for this challenging condition.

## Introduction and background

Endometriosis is a chronic and often debilitating condition where tissue similar to the lining inside the uterus (endometrium) grows outside the uterus, commonly on the pelvic peritoneum, ovaries, and fallopian tubes [1]. This ectopic tissue responds to hormonal changes during the menstrual cycle, leading to inflammation, scarring, and the formation of adhesions. The condition affects an estimated 10% of women of reproductive age globally, making it one of the most prevalent gynecological disorders [2]. The pathogenesis of endometriosis involves a complex interplay of genetic, hormonal, immunological, and environmental factors. Central to its progression are inflammatory mechanisms that perpetuate tissue proliferation and adhesion formation outside the uterus [2]. Dysregulated immune responses further contribute to the persistence of ectopic endometrial implants, exacerbating symptoms such as pelvic pain, dysmenorrhea (painful periods), dyspareunia (painful intercourse), and infertility [3].

Beyond its symptomatic impact, endometriosis significantly affects fertility. Endometrial-like tissue in abnormal locations can disrupt ovarian function, impair follicular development, and interfere with ovulation and fertilization [4]. In severe cases, extensive adhesions and scarring may lead to anatomical distortions that compromise reproductive organ function. Consequently, women with endometriosis often face challenges in conceiving naturally and may require assisted reproductive technologies to achieve pregnancy [5]. This review explores the intricate connections between inflammatory mechanisms and fertility implications in endometriosis. By synthesizing current knowledge and research findings, it seeks to elucidate how inflammation drives disease progression and influences reproductive outcomes. Understanding these underlying mechanisms is crucial for advancing diagnostic approaches, refining treatment strategies, and improving the management and quality of life for individuals affected by this complex condition. Through a comprehensive exploration, this review endeavors to contribute to the broader understanding of endometriosis and pave the way for enhanced therapeutic interventions tailored to address both its inflammatory and reproductive sequelae.

## Review

Pathophysiology of endometriosis

Normal Endometrial Physiology

The endometrium, the inner lining of the uterus, undergoes a complex series of processes essential for implantation, pregnancy maintenance, and menstruation regulation. It comprises two layers: the functional layer that sheds during menstruation and the basal layer that remains intact [6]. The endometrium is highly responsive to fluctuating estrogen (E2) and progesterone (P4). Throughout the menstrual cycle, the endometrium changes structure and function in response to these hormones. The withdrawal of progesterone triggers menstruation, leading to the detachment of the functional layer [7]. The endometrium comprises various cell types, including glandular and luminal epithelium, stromal fibroblasts, vascular endothelium, smooth muscle cells, resident and transient immune cells, and stem cells. It is characterized by its ability to undergo rapid repair without scarring or loss of function, akin to the regenerative capacity observed in a developing fetus [7]. The endometrium houses tissue-resident immune cells and experiences a cyclical influx of innate immune cells, crucial for the breakdown and repair of endometrial tissue during menstruation. Alterations in immune cell populations within the eutopic endometrium can impact other endometrial cells, potentially leading to lesion formation, persistence, and growth [8]. Menstruation and endometrial repair offer a valuable in vivo model for studying inflammation and tissue repair. A comprehensive understanding of these processes mechanisms and their disruptions is vital for enhancing patient management and developing treatments for conditions such as abnormal uterine bleeding (AUB) [8]. Recent technological advancements, including* in vitro* organoid models, co-culture systems, stem cell research, microfluidics, menstrual fluid studies, bioinformatics, big data analytics, 'omics' approaches, imaging, and artificial intelligence, are providing deeper insights into endometrial physiology and pathology [8].

Ectopic Endometrial Tissue Development

The development of ectopic endometrial tissue in endometriosis involves a complex interplay of pathophysiological mechanisms. The prevailing theory suggests that endometriosis arises from retrograde menstruation, where menstrual blood containing endometrial cells flows back through the fallopian tubes and implants on pelvic organs and the peritoneum. This theory is supported by congenital outflow tract obstructions, which can elevate the risk of developing endometriosis [3]. Ectopic endometrial lesions induce an inflammatory response by releasing inflammatory mediators, including cytokines, chemokines, and prostaglandins. This inflammatory milieu can impair oocytes, sperm, and embryos, compromising their quality and viability. Hormonal imbalances associated with endometriosis, such as elevated estrogen levels and sensitivity, along with disruptions in the estrogen-progesterone balance, can further affect follicle maturation, ovulation, and endometrial receptivity, thereby contributing to infertility [9]. Genetic predisposition and environmental factors, including exposure to endocrine-disrupting chemicals, also play a role in the development of endometriosis. A family history of the disease is a significant risk factor, and specific genetic variants have been linked to the condition. Additionally, altered pelvic anatomy and the formation of adhesions can distort pelvic structures, hindering the release and capture of oocytes and disrupting sperm motility and embryo transport through the fallopian tubes. These anatomical changes exacerbate fertility challenges in individuals with endometriosis [10].

Inflammatory Pathways Involved

The inflammatory pathways in endometriosis are intricate and multifaceted. A central feature is the elevated presence of pro-inflammatory cytokines such as IL-1β, IL-6, IL-8, IL-17, TNF-α, and COX2 in endometriotic lesions, peritoneal fluid, and serum. These cytokines drive endometriotic lesions' survival, growth, invasion, angiogenesis, and immune evasion. Concurrently, there is an altered expression of anti-inflammatory cytokines such as IL-4, IL-10, and TGF-β, which further exacerbates the inflammatory environment [11]. Immune dysregulation is also critical in the pathogenesis of endometriosis. An increased number of peritoneal macrophages and their inflammatory products contribute to chronic inflammation and oxidative stress.

Additionally, macrophage phagocytic activity is impaired, and the cytolytic function of natural killer cells is diminished. T-cell function is also disrupted, and leukocytes accumulate in ectopic lesions [12]. Hormonal regulation of inflammation plays a significant role as well. Estrogen enhances the expression and release of pro-inflammatory factors, whereas progesterone resistance contributes to ongoing inflammation. Inflammation can also influence hormonal regulation by modulating sex steroid receptors and increasing aromatase activity [11].

Furthermore, the inflammasome pathway is dysregulated in endometriosis. Components of the inflammasome, such as the NLRP3 sensor and caspase 1, are disrupted, leading to increased activation of IL-1β. The interaction between estrogen receptor β, inflammasome components, and apoptosis regulators also impairs apoptosis and promotes inflammation [11].

Clinical manifestations

Common Symptoms (Pain, Infertility, Menstrual Irregularities)

**Figure 1:**
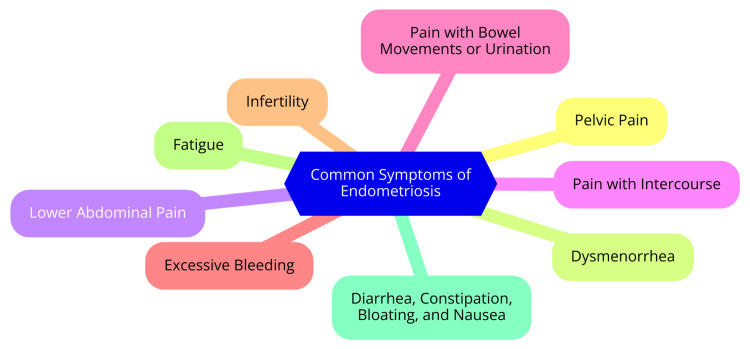
Common symptoms of endometriosis Image Credit: Dr. Sachin Rathod

Endometriosis is a complex and debilitating condition that can profoundly affect a woman's reproductive health and overall quality of life. One of the most prevalent and disruptive symptoms is chronic pelvic pain, which often intensifies during menstrual periods. This severe pain can hinder daily functioning and significantly impact well-being [1]. Infertility is another major concern for women with endometriosis. The condition is a leading cause of infertility, with women experiencing infertility being 6-8 times more likely to have endometriosis compared to their fertile counterparts. How endometriosis affects fertility is diverse, including hormonal imbalances, impaired follicle maturation, compromised embryo implantation, and altered pelvic anatomy [4]. In addition to pelvic pain and infertility, women with endometriosis frequently encounter menstrual irregularities. These can manifest as irregular periods, with menstrual flow varying heaviness and occurring at unpredictable intervals. Such irregularities can be particularly frustrating and disruptive, complicating the ability to plan around the menstrual cycle. Other associated menstrual symptoms include painful or debilitating cramps, heavy menstrual bleeding, and premenstrual spotting or bleeding between periods [13]. A thorough understanding of the complex nature of endometriosis symptoms is essential for providing comprehensive care and support to patients. By addressing the primary clinical manifestations of pelvic pain, infertility, and menstrual irregularities, healthcare providers can enhance the overall well-being and quality of life of individuals affected by this challenging condition [14]. Common symptoms of endometriosis are shown in Figure 1.

Variations in Symptom Severity and Presentation

Endometriosis can present with significant variability in symptom severity and manifestation, complicating diagnosis and management. The primary symptoms are pelvic pain and infertility. Pain associated with endometriosis can be severe and debilitating, often exacerbated during menstrual periods and after sexual intercourse. Infertility is a common complication, affecting up to 50% of women with the condition [15]. Notably, the severity of symptoms does not always correlate with the extent or stage of the disease. Some women with minimal endometriosis may experience intense pain, whereas others with extensive disease might have few or no symptoms. The pain related to endometriosis is complex and multifaceted, stemming from factors such as the growth and shedding of endometrial tissue outside the uterus, inflammation, adhesions, and nerve involvement. Although the precise mechanisms are not fully understood, research indicates that hormonal fluctuations, inflammation, and nerve sensitivity are significant contributors [16]. Symptoms can vary widely between individuals. Some women may experience heavy menstrual bleeding, while others might have no bleeding at all.

Similarly, pain levels can range from severe to mild or even absent. This variability poses challenges for both diagnosis and treatment. Endometriosis can significantly affect a woman's quality of life, leading to fatigue, reduced physical activity, and emotional distress, even in the absence of severe pain or infertility [17]. Recognizing these variations in symptom severity and presentation is crucial for delivering effective care and support to women with endometriosis. Healthcare providers need to be attuned to the diverse manifestations of the condition and collaborate closely with patients to create personalized treatment plans that address their specific needs and concerns [18].

Diagnosis

Challenges in Diagnosis

The diagnosis of endometriosis presents several significant challenges. One primary obstacle is the non-specific nature of the symptoms associated with the condition. Common symptoms such as pelvic pain, heavy menstrual bleeding, and pain during intercourse can also occur in a range of other gynecological disorders. This overlap in symptoms can delay diagnosis and appropriate treatment [19]. Another major challenge is unreliable screening tools and diagnostic tests for endometriosis. Traditional imaging techniques, including ultrasound and magnetic resonance imaging (MRI), often fail to detect endometriotic lesions accurately, particularly smaller ones. Although research is ongoing to develop alternative diagnostic methods, many of these proposed tests are not yet validated or sufficiently accurate to replace the current gold standard of laparoscopic visualization and biopsy [20]. The general lack of awareness and education about endometriosis within the healthcare community also exacerbates diagnostic challenges. Menstrual pain is often normalized and dismissed, leading to symptoms being overlooked or misinterpreted. Understanding and recognizing endometriosis among healthcare providers is essential for ensuring timely and accurate diagnoses [21].

**Figure 2:**
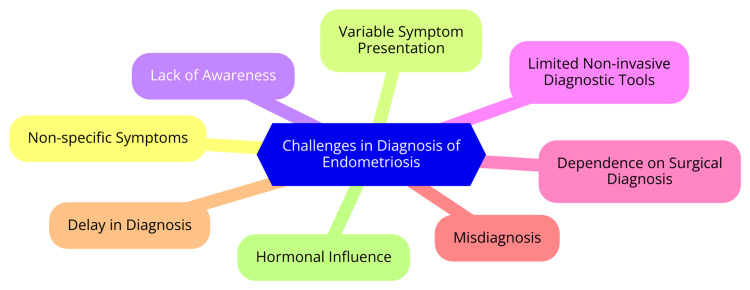
Challenges in the diagnosis of endometriosis Image Credit: Dr. Sachin Rathod

Moreover, the variability in endometriosis manifestation further complicates diagnosis. The disease may present with small, hard-to-detect lesions that cause significant symptoms. Conversely, the larger lesions may present with few or no symptoms. This variability in disease presentation adds to the complexity of diagnosis [22]. Finally, the reliance on laparoscopic surgery for a definitive diagnosis is a considerable challenge. Laparoscopy is an invasive procedure requiring general anesthesia and carries inherent risks. The necessity of this invasive surgical procedure as the gold standard for diagnosis underscores the urgent need to develop non-invasive diagnostic tools capable of accurately identifying endometriosis [23]. Challenges in the diagnosis of endometriosis are shown in Figure 2.

Diagnostic Methods

Diagnosing endometriosis can be complex due to the wide range of symptoms and the variability in lesion appearance. The diagnostic journey typically begins with a thorough clinical evaluation by an experienced clinician. The healthcare provider will gather a detailed medical history and perform a physical examination during this evaluation. Patients may report symptoms such as pelvic pain, heavy or irregular periods, and painful intercourse, which can offer clues about the presence of endometriosis. However, these symptoms are not exclusive to endometriosis and can be associated with other conditions as well.

A pelvic examination may reveal abnormalities such as cysts, nodules, or scars, but smaller endometriotic lesions may be missed during this assessment [24]. Imaging techniques play a crucial role in the diagnostic process. Transvaginal ultrasound is a relatively cost-effective and accessible method to help visualize larger endometriotic lesions, such as endometriomas (ovarian cysts). MRI provides more detailed information about the location and size of endometriotic lesions, particularly for deep infiltrating disease. Nonetheless, imaging tests cannot reliably detect all types of endometriotic lesions and cannot definitively diagnose the condition [25]. Ultimately, laparoscopy, a minimally invasive surgical procedure, is considered the gold standard for diagnosing endometriosis. The surgeon can visually inspect the pelvic cavity during laparoscopy and obtain biopsies of suspicious lesions for definitive confirmation. This procedure remains the only method for a conclusive diagnosis, as imaging tests alone cannot reliably identify all lesions [16]. Diagnostic methods for endometriosis are shown in Figure 2.

**Figure 3:**
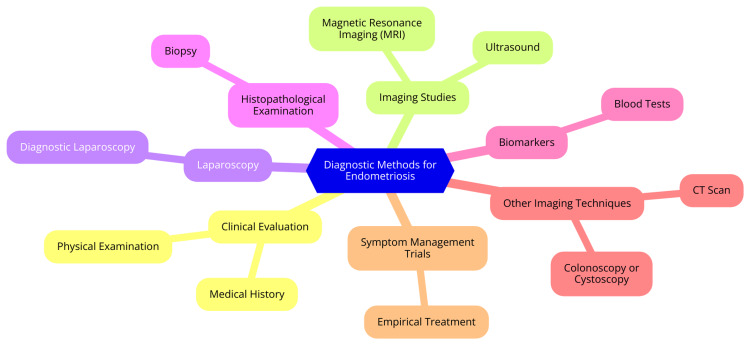
Diagnostic methods for endometriosis Image Credit: Dr. Sachin Rathod

Inflammatory mechanisms

Role of Inflammation in Endometriosis Progression

The role of inflammation in the progression of endometriosis is complex and multifaceted. Central to this condition is hormonal imbalance, particularly an increase in estrogen production. Elevated estrogen levels stimulate the enzyme cyclooxygenase-2 (COX-2), which in turn produces pro-inflammatory prostaglandin E2 (PGE2). This process exacerbates the inflammatory state, creating a self-perpetuating cycle [26]. The inflammatory environment in endometriosis is characterized by an intricate balance between pro-inflammatory cytokines, such as IL-1, IL-6, and TNF-α, and anti-inflammatory cytokines, including IL-4, IL-10, and TGF-β. These cytokines are pivotal in influencing cell proliferation, differentiation, and immune function, all of which contribute to the progression of endometriosis [27]. One key mechanism by which inflammation drives endometriosis progression is through the impairment of immune function. The inflammatory milieu can enable ectopic endometrial lesions to evade immune detection and destruction, allowing them to continue growing and invading. Anti-inflammatory cytokines may play a significant role in this immune evasion [28].

**Figure 4:**
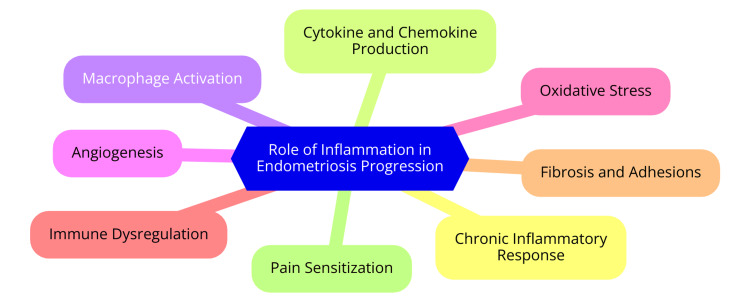
Role of inflammation in endometriosis progression Image Credit: Dr. Sachin Rathod

Additionally, inflammation fosters the growth, invasion, and vascularization of endometriotic lesions through the actions of both pro-inflammatory and anti-inflammatory cytokines. These cytokines also contribute to developing fibrosis and adhesions, worsening the condition [29]. The role of inflammation in endometriosis progression is shown in Figure 4.

Cellular and Molecular Pathways

The cellular and molecular pathways involved in endometriosis, particularly inflammation-related, are complex and intricately interconnected. One of the key pathways is the NF-κB (nuclear factor kappa B) pathway, which is a critical regulator of inflammation and immune responses. In endometriosis, the NF-κB pathway becomes activated, leading to the expression of pro-inflammatory genes and the production of cytokines such as TNF-α and IL-1β. This activation contributes to the chronic inflammation that is characteristic of endometriosis, resulting in pain, infertility, and other symptoms [30]. Another significant pathway in endometriosis is the prostaglandin pathway, especially involving prostaglandin E2 (PGE2). PGE2 is synthesized by the enzyme cyclooxygenase-2 (COX-2) and amplifies the inflammatory response by stimulating the production of additional pro-inflammatory mediators. This process exacerbates the chronic inflammation observed in endometriosis, further perpetuating the disease.

The mitogen-activated protein kinase (MAPK) pathway regulates cell proliferation and invasion in endometriosis. It interacts with other signaling pathways, such as the RAP1 pathway, to promote the development of endometriosis. Dysregulation of the MAPK pathway in endometriotic lesions contributes to endometrial cells' abnormal growth and behavior outside the uterus [31]. The transforming growth factor-β (TGF-β) pathway is another critical player in the pathogenesis of endometriosis. TGF-β is a multifunctional cytokine that governs cell growth, differentiation, and apoptosis. In endometriosis, alterations in the TGF-β pathway lead to increased fibrosis and the formation of fibrotic tissue within and around endometriotic lesions.

Additionally, the Wnt/β-catenin pathway, a key biological regulatory system, is frequently dysregulated in endometriosis. This pathway is involved in stem cell homeostasis and morphogenesis, and its aberrant activity in endometriotic lesions contributes to the establishment and progression of the disease [32]. Finally, the PI3K/Akt/mTOR pathway regulates cell growth, proliferation, and survival in endometriosis. Dysregulation of this pathway in endometriotic lesions contributes to the abnormal growth and behavior of endometrial cells outside the uterus. These pathways are interconnected and interact, creating a complex network of molecular interactions that drive the development and progression of endometriosis. Understanding these pathways is essential for developing targeted therapies to manage symptoms and improve fertility outcomes in women with endometriosis [33].

Impact on fertility

Mechanisms of Infertility in Endometriosis

Endometriosis can significantly impact fertility through multiple mechanisms. One major factor is the distortion of pelvic anatomy. Endometriotic lesions and adhesions can create anatomical distortions in the pelvis, fallopian tubes, and ovaries, which can obstruct fertility. These distortions can hinder oocyte release, ovum capture, and embryo transport, complicating fertilization [34]. Inflammatory responses triggered by endometriosis are another key mechanism affecting fertility. The presence of inflammatory cytokines, oxidative stress, and altered immune cells in the peritoneal fluid can disrupt normal reproductive function. This inflammation can damage gametes and embryos and impair endometrial receptivity, making pregnancy difficult [35]. Hormonal imbalances are also crucial in endometriosis-related infertility. Elevated estrogen levels, a common feature of endometriosis, can further exacerbate infertility. These hormonal disruptions can negatively affect ovulation, oocyte quality, and endometrial function, making conception more challenging [36]. Endometriosis can also impair ovarian function, reducing ovarian reserve and diminishing oocyte quality. This decline in ovarian function can decrease fertility and complicate the conception process. Additionally, the endometrium in women with endometriosis may be less receptive to embryo implantation, further reducing the likelihood of a successful pregnancy [37].

**Figure 5:**
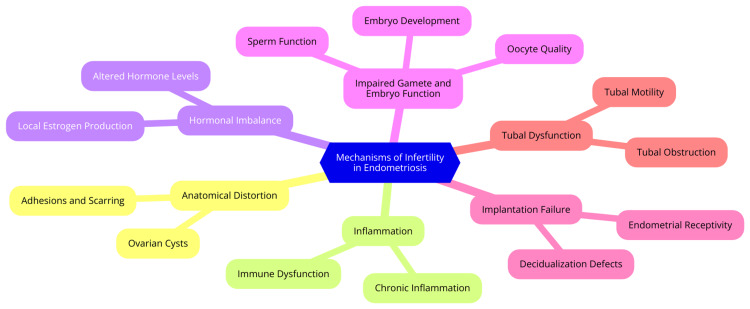
Mechanisms of infertility in endometriosis Image Credit: Dr. Sachin Rathod

While the link between endometriosis and infertility is well-established, the precise mechanisms are not yet fully understood. Ongoing research investigates the interplay between inflammation, hormonal imbalances, and reproductive processes to clarify how endometriosis affects fertility. A deeper understanding of these mechanisms is essential for developing effective treatments and improving fertility outcomes for women with endometriosis [38]. Mechanisms of infertility in endometriosis are shown in Figure 5.

Effects on Ovarian Reserve and Follicular Development

Endometriosis can have a profound impact on ovarian reserve and follicular development, leading to reduced fertility. The presence of endometriomas, or ovarian cysts, is linked to decreased ovarian reserve, as evidenced by lower levels of anti-Müllerian hormone (AMH) and a reduced antral follicle count (AFC) in women with endometriosis compared to healthy controls. This decline in ovarian reserve significantly contributes to the infertility observed in many women with the condition [39]. The mechanisms through which endometriosis affects ovarian reserve are complex. Inflammation and oxidative stress within the ovarian environment can disrupt normal ovarian function and steroid hormone production.

Additionally, the hyperactivation of primordial follicles can lead to premature depletion of the follicular pool, further diminishing ovarian reserve. Direct damage to ovarian tissue caused by endometriomas also contributes to reducing ovarian reserve [40]. Surgical removal of endometriomas can further exacerbate the decline in ovarian reserve, especially when techniques such as bipolar cauterization are employed, which can cause thermal damage to the ovarian tissue. This underscores the importance of careful surgical management and the consideration of fertility preservation options for women with endometriosis. The impact of endometriosis on ovarian reserve and follicular development highlights the need for early diagnosis and effective treatment strategies to preserve fertility and enhance reproductive outcomes for these patients [41].

Management Strategies For Fertility Preservation

Surgical intervention plays a vital role in managing fertility preservation for women with endometriosis. Laparoscopic or laparotomic removal of endometriotic lesions and adhesions can enhance fertility outcomes by restoring normal pelvic anatomy. This approach addresses the anatomical distortions caused by endometriosis, which can obstruct fertility [42]. Assisted reproductive technologies (ART) are also crucial for women experiencing infertility due to endometriosis. Techniques such as *in vitro* fertilization (IVF) can effectively overcome the disease's challenges. By bypassing the affected reproductive organs and facilitating fertilization directly, ART offers a viable path to pregnancy despite the underlying condition [43]. Ovarian tissue cryopreservation is another strategy for fertility preservation. This method involves removing and freezing ovarian tissue, which can later be transplanted into the patient. This approach is particularly advantageous for young women with endometriosis, as it helps preserve ovarian function and fertility potential [44]. *In vitro* maturation (IVM) of oocytes is an emerging technique that could benefit patients with endometriosis. IVM allows the maturation of immature oocytes retrieved without the need for ovarian stimulation, which may be advantageous for women concerned about the effects of hormonal therapies on their condition [45]. While medical therapies that suppress fertility are currently not recommended for treating endometriosis-associated infertility, future research may yield non-hormonal treatments targeting the inflammatory pathways involved in the disease. Such developments could provide additional options for women seeking fertility preservation [4]. Managing endometriosis-related infertility requires a comprehensive approach that addresses the multifaceted nature of the disease, including anatomical distortions, inflammation, hormonal imbalances, and impaired ovarian function. By integrating surgical, ART, and emerging fertility preservation techniques, healthcare providers can offer women with endometriosis the best possible chance of preserving their fertility and achieving successful pregnancies [46].

Treatment approaches

Medical Management (Hormonal Therapies, NSAIDs)

Medical management of endometriosis encompasses various hormonal therapies and non-steroidal anti-inflammatory drugs (NSAIDs) aimed at alleviating symptoms and reducing pain. Gonadotropin-releasing hormone (GnRH) agonists are a class of hormonal treatments that suppress ovarian hormone production, which can mitigate symptoms and pain associated with endometriosis. These medications are typically used for moderate to severe cases and induce temporary chemical menopause, which may lead to side effects such as decreased bone density and menopausal symptoms [18]. Progestins are another hormonal therapy option that can halt menstrual periods and slow the growth of endometrial tissue. Often used in conjunction with other treatments, progestins can provide significant pain relief for many women with endometriosis. Combined oral contraceptive pills (COCPs) are also employed to regulate hormone levels and alleviate endometriosis symptoms. Continuous use of COCPs can prevent menstruation and associated discomfort while offering contraceptive benefits [47]. Aromatase inhibitors are prescribed for cases of refractory or recurrent endometriosis. These medications work by inhibiting the conversion of androgens to estrogens, thus lowering estrogen levels and potentially slowing endometriosis progression. NSAIDs, such as ibuprofen and naproxen, are frequently used to manage pain related to endometriosis. They function by reducing inflammation and providing temporary relief. However, it is essential to recognize that hormonal therapies are generally unsuitable for women seeking pregnancy, as they may impact ovulation. Women with endometriosis who wish to conceive may need to explore alternative treatments or surgical options.

Additionally, hormonal therapies can lead to significant side effects, including bone density loss, menopausal symptoms, and an increased risk of osteoporosis. NSAIDs can also cause gastrointestinal issues and other side effects [48]. The choice of treatment for endometriosis depends on the individual’s symptoms, fertility goals, and personal preferences. While medical management can effectively control symptoms and reduce pain, it is crucial to consider the potential benefits alongside the risks and side effects of each treatment option [49].

Surgical Options (Laparoscopic Excision, Ablation)

Laparoscopic surgery is the primary surgical treatment for endometriosis, employing two main techniques: excision and ablation. The choice of technique depends on factors such as the size, location, and depth of the endometriotic lesions. Laparoscopic excision involves the complete removal of endometriotic tissue using cutting techniques. This method allows for thorough extraction of the lesions, which can then be examined microscopically to confirm the diagnosis. Studies have demonstrated that excision is more effective than a placebo in reducing pain and improving the quality of life for women with endometriosis across all stages. When both excision and ablation are viable options, excision is generally preferred for its superior outcomes in pain relief [50].

In contrast, laparoscopic ablation involves destroying endometriosis cells using heat (diathermy) or laser. This technique can address multiple endometriotic spots throughout the pelvis. While ablation may be used with excision depending on lesion characteristics, it does not allow for tissue diagnosis. It is often less effective in providing long-term pain relief than excision [51]. Both techniques are minimally invasive, involving small abdominal incisions to insert a camera and surgical instruments. This approach typically results in faster recovery compared to open surgery. The decision between excision and ablation ultimately depends on the specific attributes of the endometriotic lesions and the surgeon’s expertise [52].

Emerging Therapies and Future Directions (Immunomodulators, Targeted Therapies)

Endometriosis is a multifaceted disease characterized by the presence of endometrial tissue outside the uterus, which can have a significant impact on fertility. Emerging therapies and future directions in the treatment of endometriosis are increasingly focusing on immunomodulators and targeted therapies. Immunomodulators, such as tumor necrosis factor-alpha (TNF-α) blockers, have shown promise in reducing endometriosis-associated pain and lesion size. Additionally, inflammatory cytokines such as interleukin-1β (IL-1β) and interleukin-6 (IL-6) are being investigated as potential targets for new therapies. Targeted therapies such as antiangiogenic agents that inhibit vascular endothelial growth factor (VEGF) are also under exploration for their potential to reduce the growth and spread of endometriotic lesions [9]. There is a growing emphasis on developing non-hormonal treatments for endometriosis, given that current hormonal therapies can have significant side effects and do not provide a cure. Research actively explores strategies to target inflammatory pathways, oxidative stress, and other molecular mechanisms involved in endometriosis pathogenesis.

Moreover, combination therapies that integrate hormonal treatments, immunomodulators, and targeted therapies may offer a more comprehensive approach to managing endometriosis, aiming to address its multifactorial nature and improve long-term outcomes [53]. Future advancements may also involve identifying specific biomarkers or genetic factors associated with endometriosis to enable more personalized treatment approaches. Tailoring therapies to individual patient characteristics and disease profiles could lead to more precise and effective interventions. This personalized medicine approach can potentially revolutionize the treatment of endometriosis, offering more targeted and effective solutions. The future of endometriosis treatment is promising, focusing on immunomodulators, targeted therapies, non-hormonal treatments, combination therapies, and personalized medicine [54].

Quality of life and psychological impact

Challenges in Managing Chronic Pain and Fertility Concerns

Chronic pain conditions affecting the female reproductive system, such as endometriosis, pelvic adhesions, or uterine fibroids, can complicate conception. Treatments for these conditions, including hormonal birth control or hysterectomy, may significantly reduce or eliminate the ability to conceive or maintain a pregnancy. In men, chronic musculoskeletal pain has been associated with adverse effects on sperm concentration, motility, and the percentage of hyperactivated sperm, potentially impacting male fertility [55]. The management of chronic pain during pregnancy is an area that remains underexplored, and obstetric providers often have limited formal training in this domain. Current information predominantly addresses the management of preexisting chronic pain conditions during pregnancy, with insufficient data on chronic pain disorders that may develop during pregnancy. There is a pressing need for evidence-based guidelines to assist clinicians in managing pregnant women with chronic pain disorders and to clarify the roles of the clinical care team [56]. Extended periods of bed rest can exacerbate chronic pain by leading to stiffness, muscle and bone weakness, poor sleep, loneliness, depression, and intensified pain. A more effective approach involves a combination of exercise, continued work, physical therapy, and judicious use of painkillers to alleviate pain and promote activity. Selecting appropriate exercises that do not excessively strain the body, such as flexibility and sitting, is crucial for effectively managing chronic pain [57].

Impact on Mental Health and Well-Being

Endometriosis is a chronic and debilitating condition that not only inflicts physical pain and discomfort but also significantly affects a woman's mental health and overall well-being. Research consistently reveals that women with endometriosis face a higher risk of developing depression and anxiety compared to those without the condition. The prevalence of depressive symptoms among endometriosis patients ranges from 9.8 to 98.5%, while anxiety symptoms are reported in 11.5 to 87.5% of cases [1]. Several factors contribute to the mental health impact of endometriosis. Chronic pelvic pain, a hallmark of the condition, is a major source of stress and can lead to feelings of helplessness and frustration. Infertility, which often accompanies endometriosis, can profoundly affect a woman’s mental health, resulting in grief, loss, and feelings of inadequacy.

Additionally, diagnostic delays-common due to insufficient awareness and understanding of endometriosis-can exacerbate the psychological burden, leading to feelings of being dismissed or invalidated by healthcare providers [58]. Endometriosis also significantly affects a woman’s health-related quality of life (HRQoL), impacting physical, social, and emotional domains. Key factors contributing to reduced quality of life include pelvic pain, sexual dysfunction, and infertility. Longer diagnostic delays are linked to poorer HRQoL, underscoring the need for prompt diagnosis and treatment. Other factors, such as acceptance of the illness, impacts on relationships, loss of control over symptoms, and maladaptive coping strategies, further contribute to impaired quality of life [59]. Despite these challenges, interventions are available to improve outcomes. Both medical and surgical treatments, along with psychological interventions such as cognitive-behavioral therapy, have been shown to enhance the quality of life and mental health outcomes for endometriosis patients. Integrating psychological assessment and support into a multidisciplinary approach is essential for addressing the complex biopsychosocial impact of endometriosis [60].

## Conclusions

In conclusion, endometriosis presents a multifaceted challenge characterized by its inflammatory nature and profound implications for fertility. The inflammatory mechanisms driving the pathogenesis of this condition contribute significantly to its chronic pain and systemic effects. Moreover, the disorder's impact on fertility underscores the need for early diagnosis and targeted management strategies to preserve reproductive health. Advances in understanding the immunological and molecular pathways involved in endometriosis have paved the way for innovative therapeutic approaches, including hormonal therapies, surgical interventions, and emerging treatments to modulate immune responses. However, further research is essential to unravel the complexities of endometriosis fully and to develop personalized treatments that address both symptomatic relief and fertility restoration. By continuing to explore these avenues, healthcare providers can better support individuals with endometriosis, offering hope for improved outcomes and enhanced quality of life for patients worldwide.
